# Closed-loop motor imagery EEG simulation for brain-computer interfaces

**DOI:** 10.3389/fnhum.2022.951591

**Published:** 2022-08-17

**Authors:** Hyonyoung Shin, Daniel Suma, Bin He

**Affiliations:** Department of Biomedical Engineering, Carnegie Mellon University, Pittsburgh, PA, United States

**Keywords:** brain-computer interfaces, EEG simulation, closed-loop systems, motor imagery, sensorimotor rhythm

## Abstract

In a brain-computer interface (BCI) system, the testing of decoding algorithms, tasks, and their parameters is critical for optimizing performance. However, conducting human experiments can be costly and time-consuming, especially when investigating broad sets of parameters. Attempts to utilize previously collected data in offline analysis lack a co-adaptive feedback loop between the system and the user present online, limiting the applicability of the conclusions obtained to real-world uses of BCI. As such, a number of studies have attempted to address this cost-wise middle ground between offline and live experimentation with real-time neural activity simulators. We present one such system which generates motor imagery electroencephalography (EEG) *via* forward modeling and novel motor intention encoding models for conducting sensorimotor rhythm (SMR)-based continuous cursor control experiments in a closed-loop setting. We use the proposed simulator with 10 healthy human subjects to test the effect of three decoder and task parameters across 10 different values. Our simulated approach produces similar statistical conclusions to those produced during parallel, paired, online experimentation, but in 55% of the time. Notably, both online and simulated experimentation expressed a positive effect of cursor velocity limit on performance regardless of subject average performance, supporting the idea of relaxing constraints on cursor gain in online continuous cursor control. We demonstrate the merits of our closed-loop motor imagery EEG simulation, and provide an open-source framework to the community for closed-loop SMR-based BCI studies in the future. All code including the simulator have been made available on GitHub.

## Introduction

Brain-computer interfaces (BCIs), particularly ones that can decode motor intention, are the subject of active research due to their potential as neural prosthetic systems capable of improving quality of life for patients suffering from various motor function impairing conditions such as spinal cord injury, amyotrophic lateral sclerosis, and stroke (Anderson, 2004; He et al., 2020). One well-established approach is sensorimotor rhythm (SMR)-based motor imagery BCIs, which allow users to control the movement of an agent in the physical or virtual world by detecting and decoding SMR patterns associated with real and imagined movements (Wolpaw and McFarland, 1994; Yuan and He, 2014; He et al., 2015).

Generally in a BCI system, the performance of the neural activity decoder is critical and a significant proportion of BCI research is dedicated to maximizing it through feature engineering, signal processing and machine learning, as well as optimization of the parameters and hyperparameters these methods depend on. A large proportion of such research evaluates decoder designs and parameters on pre-recorded datasets in an offline environment (Chavarriaga et al., 2016). In contrast, a BCI ultimately aims to provide real-time decoding of neural activity and enacting of the decoder output [for example, as a physical movement of a computer cursor (Wolpaw and McFarland, 1994), a drone (LaFleur et al., 2013), or a robotic limb (Meng et al., 2016; Edelman et al., 2019)], which serves as feedback for the central nervous system of the user to adjust its output (i.e., BCI input) accordingly. A functional BCI is fundamentally defined by this bidirectionally adaptive relationship between decoder and user, and almost any example of a BCI application, clinical or non-clinical, is indeed found in the domain of online closed-loop usage, rather than an offline one. Importantly, several studies have successfully highlighted differences in conclusions obtained from offline and online analyses of the same range of decoder parameters being tested (Cunningham et al., 2011; Chavarriaga et al., 2016). Some studies have recognized this problem and have attempted to incorporate online control dynamics into offline evaluations by predicting decoder output in small bins of time (Dose et al., 2018; He and Wu, 2020; Stieger et al., 2021b). However, they do not address the fundamental problem with offline analyses that the subject is absent from the feedback loop. This lack of inclusion results in evaluations that fail to take into account the effect of co-adaptation in the performance of the BCI system, as well as on the effect of decoder and task parameters on subject control behavior. This is especially the case with SMR-based BCIs in which there are large variances in each subject's ability, method and rate of learning to modulate their SMRs to accomplish a BCI task. Therefore, it can be said that while offline analysis plays an important role in certain cases or development phases of a BCI system, it is desirable to evaluate BCI decoders as well as tasks (which are invariably linked to the decoder in that a decoder's output is in terms of a change in the task space) in an online, closed-loop setting.

We hypothesize that the relative abundance of offline BCI evaluation studies ultimately stems from the cost and hassle associated with running live BCI experiments. Indeed, to do live experiments, one must own, regularly maintain, and be trained in using signal acquisition equipment(s), recruit human subjects, train them how to do BCI control in the specific experimental paradigm(s) being used (Roc et al., 2021), ensure their safety, deal with confounding factors such as intra-/inter-session/subject (Scherer et al., 2012; Saha and Baumert, 2020) variability as well as various recording artifacts, and perhaps most importantly, spend a lot of time compared to offline analyses, which is a disadvantage from both man-hours and time-to-data perspectives. Cunningham et al. (2011) has previously proposed closed-loop neural activity simulations as a cost-wise middle ground between online and offline experimentation, and in turn, their online prosthesis simulator (OPS) and the brain-machine interface simulator by Kwon and Kim (2010) have attempted to address this important gap. In the former, artificial spiking activity was generated based on the kinematics of subject reaches in a hidden 3D volume, which was decoded by a Kalman filter to provide visual reaching feedback to the subject. In the latter, artificial spiking activity was generated from computer mouse movements through a linear Gaussian model for a discrete reach task paradigm in a 2D space.

**Figure 1:**
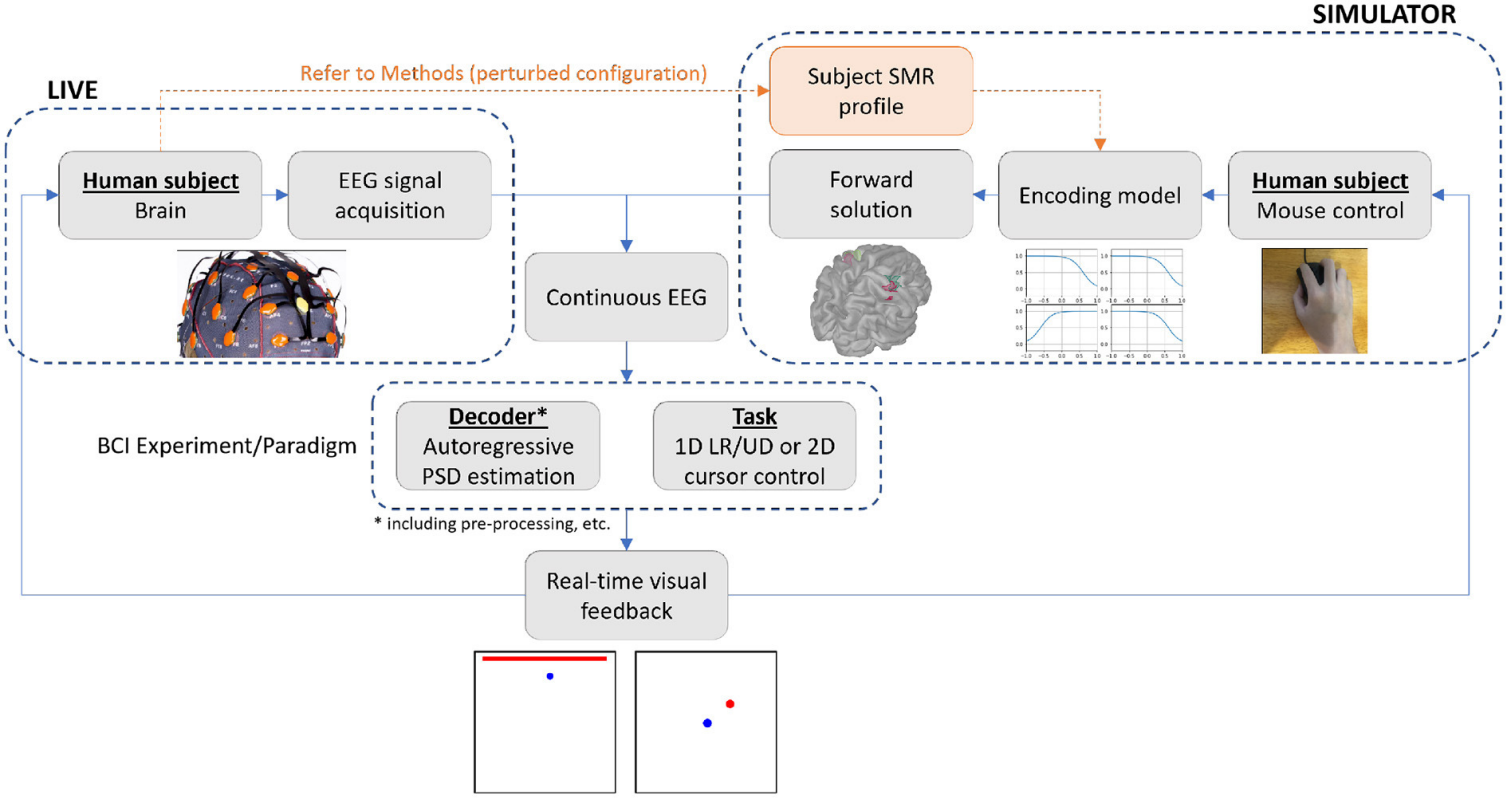
Overview of the live and simulator experiments. The simulator allows for the running of a closed-loop BCI experiment *via* real-time generation of motor imagery EEG, using subject's naturalistic mouse movements as input to represent motor intention. To verify the proposed simulator's capability to replace and/or support live experimentation, we ran the identical experimental paradigm of 1D LR center-out reaching on 10 healthy human subjects in both the live and simulated environments, and analyzed their performance against changes in selected decoder and task parameters.

To the best of our knowledge, the two studies mentioned above are the only examples of original closed-loop BCI simulators. Notably, both are designed to generate artificial spike trains and are therefore only suitable for testing decoders of spike trains, which are also utilized for invasive BCI decoders. Furthermore, neither of them is open source, making it difficult to adopt these closed-loop simulation methods in specific experiments. To address the unfulfilled cost-wise middle ground in motor imagery EEG BCI experimentation, we have developed a closed-loop BCI simulator which allows the conduction of various types of motor imagery EEG-based BCI experiments through the real-time generation of motor imagery EEG as a function of intuitive and naturalistic human subject input. We demonstrate, through a parallel parameter investigation in live and simulated environments (Figure 1), that the proposed simulator can serve as a rapid prototyping testbed for BCI decoder and task designs.

Importantly, closed-loop BCI simulators are distinguished from the relatively abundant offline simulation methods of neural activity data (Lotte, 2011; Aine et al., 2012; Krol et al., 2018; Dinarès-Ferran et al., 2018; Lindgren et al., 2018; Zhang and Liu, 2018; Aznan et al., 2019; Barzegaran et al., 2019; Fahimi et al., 2021; Kunanbayev et al., 2021; Ko et al., 2022) which serve important but different purposes [mainly for neural activity data augmentation to improve BCI decoder training and/or testing (Marturano et al., 2020; Ramírez Torres and Daly, 2021), or, more rarely, to supplement the development and evaluation of source modeling methods (Aine et al., 2012; Gramfort et al., 2013)].

In contrast, closed-loop BCI simulators are focused on providing a controlled environment for testing BCI system parameters in a specific experimental context while preserving the aspect of feedback control which is lost in offline analyses. Although this aspect, combined with the reduced time and cost compared to live experimentation, is one of the main motivations for the development of such simulators, there are other merits to using closed-loop BCI experiment simulations when compared to live BCI experimentation which are often overlooked. Here we suggest a few of them. Firstly, due to the fact that the neural activity is generated by a well-defined encoding model that maps intention input to neural activity, the effect of parameters on metrics of interest (such as but not limited to decoding performance) can safely be attributed to the changes in the parameters themselves, rather than subject-to-subject or session-to-session variability in subject behavior or recording. In other words, a closed-loop BCI simulator may be used to observe the performance ceiling of a BCI system, and assess algorithms and system design choices. Secondly, by using a pre-defined encoding model, human subjects who are unable or take longer to develop BCI control skills (often termed “BCI illiterate” subjects) are able to participate in simulated experiments without restrictions. Lastly, a ground truth of the subject's intention is captured over every time sample of the experiment as an input into the system: this is a unique type of data that cannot be collected from live BCI experiments, although it can be estimated by making assumptions—for example, by assuming that the shortest vector between a cursor and a target is the subject's motor intention in a 2D motor imagery paradigm (as done in Gilja et al., 2012; Willett et al., 2019), although this may not actually be the control policy/behavior adopted by subjects (see Section Discussion in Suma et al., 2020).

As this work, to the best of our knowledge, is the first closed-loop motor imagery EEG simulated experimental study for continuous cursor control paradigms (Edelman et al., 2019), we first set out to define the design philosophy for the simulation method. The closed-loop design of the simulator necessitates the generation of the EEG to: (1) be sufficiently simple to be computed in real-time, and (2) result in EEG similar enough in features to real motor imagery EEG such that an online decoder can be plugged in and still result in reasonable prediction of motor intention. In this sense, the EEG generator part of the simulator can indeed be viewed as a kind of a human simulator for the purposes of a specific BCI experimental paradigm. Broadly, there would be two possible approaches to EEG simulation that would fulfill both of these requirements. One approach would be to prepare large sets of multi-class EEG epochs according to the intention class (e.g., in motor imagery: left/right/up/down (L/R/U/D), or potentially finer classes that include non-cardinal directions), using pre-recorded and labeled data, offline generative models, or a mix of both. Then, the system would continuously output sequences of these pre-generated and pre-labeled epochs depending on the input class (intended movement direction). Although this would certainly work in streaming intention-dependent EEG in an online setting, we decided against this approach as it would not necessarily result in EEG that contains information about the intensity of the intention, which meant that continuous cursor (or more generally, agent) control task paradigms would not work with such a simulation without making undesirable assumptions such as constant/randomized/discretized cursor velocity. The alternative is to attempt to parameterize typical EEG signals in some systematic and continuous manner, and map the intended movement intention into those parameters. Such an approach would inherently result in continuous variation of the synthetic EEG according to the input movement intention with the intensity information preserved. The two challenges with this approach would then be to come up with a reasonable system to parameterize EEG signals, as well as a reasonable encoding model to map movement intention to these EEG-controlling parameters. It can be said that addressing these challenges is critically linked to the realism and practicality of the resulting closed-loop EEG simulation.

## Methods

**Figure 2:**
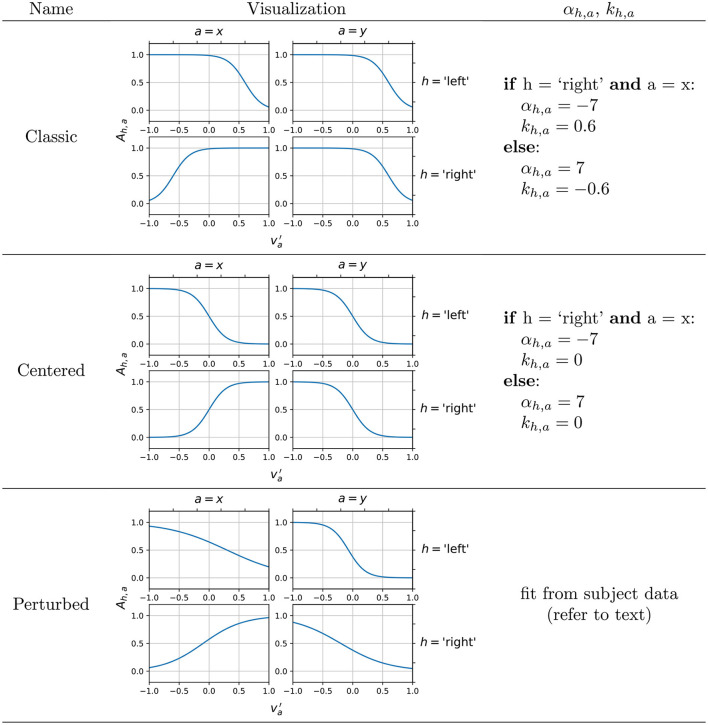
Example configurations of the encoding model, providing different mapping of the subject intention input $v_{a}^{\prime }$ to amplitude modulation factors *A*_*h,a*_. In the perturbed configuration, previously recorded subject EEG is used to fit the encoding functions, and an example output is shown here.

With the above in mind, we decided on a forward solution-based generative approach inspired by offline EEG simulation methods such as SEREEGA (Krol et al., 2018) and EEGSourceSim (Barzegaran et al., 2019) in that some customized time-series activation signals assigned to source locations in a 3D head model are projected onto the scalp through a lead field matrix. The lead field, which can be accurately estimated using modern numerical solutions, dictates how the source-level activation signals, given their source locations and orientations, are detected as scalp-level potentials at each electrode location (He et al., 2018). However unlike offline simulation methods, our closed-loop simulator automatically controls the shape of the activation signals using the subject's computer mouse control as an input representing their intuitive real-time motor intention. This is done *via* a uniquely defined, customizable (potentially subject-specific, refer to Methods Section on Perturbed configurations) encoding model which maps recorded cursor velocities to a set of amplitude modulation factors that determines the maximum normalized amplitude of the source-level activation signal. The encoding model in our case is defined simply as a set of sigmoid functions (Figure 2), the number of which depends on the motor imagery paradigm being used: two functions for 1D, four functions for 2D. We chose the sigmoid over other commonly used activation functions (such as a linear unit) for two reasons: first, we wanted tunable, generalizable functions that could transition into different configurations (see Figure 2 and Section on Neural activity encoding model), so that we could tune the effects and extents of contralateral desynchronization and ipsilateral synchronization in the simulation (discussed in Section Classic vs. centered configurations). Second, the sigmoid function captures well the extent of motor imagery plateauing at very high and very low distances to target.

To validate our EEG BCI simulator as a method capable of replacing or supplementing live experimentation in certain contexts, a comparison study (overview in Figure 1) was designed to test arrays of several parameters of interest (Table 1) in the simple 1D left/right (LR) motor imagery cursor control paradigm. These parameters were Z-score normalization bin width (BW), number of trials for control coefficient calculation (NT), and maximum cursor velocity (CV) (detailed in Methods Section on EEG acquisition and online signal processing). We picked this relatively simple experimental paradigm to minimize variables that could affect this initial evaluation of the simulator, while the parameters to be tested were carefully chosen to reflect unexplored optimizations in the motor imagery cursor control paradigm that are of interest. Although the evaluation in this study was done in the 1D motor imagery paradigm, the EEG BCI simulator was initially developed for 2D motor imagery continuous cursor control and was used to identify a non-significant effect of maximum cursor velocity relative to maximum target velocity in the 2D continuous cursor pursuit paradigm during our previous study (Shin et al., 2021). We also use the 2D LRUD center-out discrete task paradigm in this study as part of an evaluation of our 2D motor imagery encoding models (see Methods Section on Neural activity encoding model).

**Table 1:** Decoder and task parameters tested in live and simulated BCI.

**Parameter (and notation)**	**Domain**	**Range (default in bold)**
Z-score normalization bin width (BW)	Decoder	30, **60**, 90, 120s
No. of trials for control coefficient calculation (NT)	Decoder	**0**, 24, 48 trials
Maximum cursor velocity (CV)	Task	200, **250**, 300, 350 pixels/s

###  Experimental design

#### Trial structure

Ten healthy human subjects (average age 23.8 ± 3.4, 9 right-handed, 6 female) participated in one live and one simulated session each, which shared the same trial structure that is described here. Each session consisted of 10 runs of 24 1D LR center-out discrete trials block-wise randomly distributed in a pairwise fashion. Each run used a specific parameter value from Table 1, except the “NT = 0 trials” run which was jointly represented by the “BW = 60 s (default)” run, thus forming 10 runs (4 + 2 + 4). (Refer to the next section on EEG acquisition and online signal processing for a description of each of these parameters in the context of the experiment.) With a particular parameter being used as the independent variable, the other parameters not being used were fixed at a default value (Table 1). The order in which the parameter values were tested was randomized within each parameter type. Each trial began with a 3 s rest period, followed by a 2 s preparation period, in which the target was shown to the subject. Subjects were then provided up to 6 s to move the cursor to hit the target, by either modulating their neural activity (live experiment) or moving a physical computer mouse with their dominant hand (simulated experiment). If the cursor had not reached a target within the 6 s feedback control period, the trial was labeled as a timeout trial and was aborted with no target selected. Therefore, a trial had three possible outcomes: hitting the correct target (a hit), hitting the incorrect target (a miss), or timeout.

For the simulated experiment, 4 of 10 subjects participated in the experiment at home using their own hardware which were screened in advance to ensure that the simulator software ran and behaved as expected, and that each subject would participate in a valid environment. In both the live and simulated experiments, the times at which each run of the experiment began and ended were recorded digitally.

#### EEG acquisition and online signal processing

In the live experiment, EEG was acquired at a sampling rate of 250 Hz and a sensitivity of ±750 mV with the g.Nautilus RESEARCH 16 g.SAHARA (g.tec medical engineering GmbH, Austria) system connected to the BCI2000 (Schalk et al., 2004) module, which timed the experiment and visualized the task (targets and cursor feedback). In the simulated experiment, the subjects used a computer mouse on a flat office desk space of at least 25 inches width to attempt to continuously control the cursor on the screen. The mouse movements were first converted to synthetic EEG in real-time (see Section on Neural activity encoding model), which was decoded into actual cursor movements for display on the screen by a custom Python software (i.e., the simulator).

At each time sample *t*_0_, in both experiments, the EEG was pre-processed (Notch filtered 58–62 Hz, bandpass FIR filtered 2–60 Hz) and decoded online using Burg autoregressive power estimation to evaluate the alpha band (8–12 Hz) powers *PSD*_*C*3_ and *PSD*_*C*4_ at the small spatial Laplacian-filtered (McFarland et al., 1997) C3 and C4 channels, respectively (C3 neighbors: F3, T7, Cz, P3; C4 neighbors: F4, T8, Cz, P4). The raw horizontal and vertical control signals *C*_*x*_ and *C*_*y*_ were then evaluated as follows:


(1) $$\begin{array}{c} \begin{array}{cc} C_{x,t_{0}} & =PSD_{C4,\;t_{0}}-PSD_{C3,\;t_{0}}\\ C_{y,t_{0}} & =-PSD_{C4,\;t_{0}}-PSD_{C3,\;t_{0}} \end{array} \end{array}$$

*C*_*x*_ and *C*_*y*_ are then Z-scored against their own history during the past BW seconds' worth of time samples in online control, such that the resulting signal would have zero mean and unit variance. BW in Equation (2) is the normalization bin width which is one of the parameters varied in this comparison study (Table 1).


$$\begin{array}{lll} C_{a,t_{0}}^{\prime } & = & \frac{C_{a,t_{0}}-{\bar{C}}_{a,t_{0}-BW:t_{0}-1}}{\sigma_{C_{a,t_{0}-BW:t_{0}-1}}}, \end{array}$$

(2) $$\begin{array}{c} \text{where }a\text{ is the axis of control}\;x\;\text{(horizontal) or}\;y\text{ (vertical).} \end{array}$$

The first trial of each run was utilized for the calibration of the normalization bin *C*_*a*,_*t*__0_−*BW*:*t*_0_−1_, in which the subject attempted to control the cursor as per normal to “fill" the normalization bin with statistically representative raw control signals.

This normalized control signal is then directly proportional (by a constant system-dependent scaling factor *S*) to the cursor velocity to be applied at *t*_0_, in pixels:


(3) $$\begin{array}{c} \begin{array}{cc} v_{x,t_{0}} & =SC_{x,t_{0}}^{\prime }\\ v_{y,t_{0}} & =SC_{y,t_{0}}^{\prime } \end{array} \end{array}$$

However, a maximum cursor velocity CV may be enforced as a simple nonlinear threshold such that $||\vec{v}||\leq CV$; this is one of the parameters varied in this comparison study (Table 1). At the end of a run, we can choose to skip the initialization and re-calibration of the normalization bin *C*_*a*,_*t*__0_−*BW*:*t*_0_−1_ for the next run and instead make the normalization bin at the end of this run's 24 trials carry over to the next run's *t* = 0. The number of trials whose information is carried forward by this process, which is in multiples of 24, is NT, one of the parameters varied in this comparison study (Table 1). The cursor position (*x, y*) on the screen is then updated by the velocities (multiplied by a value of 1 update frame):


(4) $$\begin{array}{c} \begin{array}{c} x_{t_{0}}=x_{t_{0}-1}+v_{x,t_{0}}\\ y_{t_{0}}=y_{t_{0}-1}+v_{y,t_{0}} \end{array} \end{array}$$

#### Performance comparison

Here we define the performance metrics used in this study to represent different aspects of BCI task performance quantitatively. Calculation of performance metrics as well as the subsequent statistical analysis between live and simulated conditions were done using custom MATLAB (MathWorks Inc., MA, USA) scripts, with dependencies on EEGLAB (Delorme and Makeig, 2004). Percentage trials correct (PTC) is the proportion of total targets presented in a run that was successfully hit by the subject-controlled cursor within the 6 s feedback control period. Average decision time is the average time elapsed from the start of the feedback control period to the end of the feedback control period in each trial; a lower value is better. Average integrated distance to target in a run is the average distance of the cursor to the target during the feedback control duration; a lower value is better.

In all of the above performance metrics, all trials, including timeout trials, were included in their calculation. For example, PTC, the proportion of all trials that were correct hits (LaFleur et al., 2013), is used here instead of PVC (percentage valid correct), which is the proportion of non-timeout trials that were correct hits. Similarly, rather than calculating average hit time by averaging the duration of hit trials, average decision time is used where timeout trials were included as data points of 6 s to ensure that the metric fully represents the reduction in information transfer rate due to timeout trials. The calculation of average integrated distance to target in each run also included all trials to ensure that the metric fully represents poor BCI task performance observed in non-hit trials. We consider that this inclusion of all trials is a fair choice in evaluating the effect of our decoder and task parameters on BCI performance, as the effect of these parameters on the frequency of aborts and timeouts has not been established yet.

Next, we compared the performance vs. parameters trends observed from the live and simulated experiments. To determine if the simulator reached the same conclusion on the null hypotheses in each performance vs. parameter relationship, we fit an ordinary least squares linear regression on each live and simulated trend, and obtained the statistical significance of each linear fit term.

###  Generation of synthetic motor imagery EEG in real-time

To be comprehensive, the simulation process for a single time sample in the 2D motor imagery paradigm is described.

#### Neural activity encoding model

##### Input processing and formulation of encoding model

First, the 2D input cursor velocity $\vec{v}$ is scaled by a constant parameter *S* such that its length is at most 1:


(5) $$\begin{array}{c} {\vec{v}}^{\prime }=S\vec{v},\;|\vec{v}|\leq 1 \end{array}$$

Next, four pre-defined sigmoid functions, each corresponding to a unique pair between the two hemispheres (*h*) of the brain (left and right) and the two axes (*a*) of intended control (horizontal and vertical), are used to convert the scaled velocity ${\vec{v}}^{\prime }$ into *A*_*h,a*_ values: *A*_left,*x*_, *A*_left,*y*_, *A*_right,*x*_, and *A*_right,*y*_. In general,


(6) $$\begin{array}{c} A_{h,a}=\frac{1}{1+e^{\alpha_{h,a}\left(v_{a}^{\prime }+k_{h,a}\right)}} \end{array}$$

The gain α_*h,a*_ and the offset *k*_*h,a*_ were configured manually according to the boundary conditions reasoned below, and Figure 2 shows some example configurations that work well for the 2D motor imagery paradigm. The specific values of the gain and the offset were obtained through a qualitative calibration to ensure the output of reasonable control signals. The classic configuration serves as a default for our simulator as its design is informed by the following well-established concepts:

The presence of non-zero offsets *k*_*h,a*_, which cause $v_{a}^{\prime }=0$ (i.e., no intention input in control axis *a*) to be modeled as *A*_*h,a*_ ≈ 1 (i.e., no attenuation of amplitude in source-level activation), is based on the fact that contralateral alpha desynchronization is observed during motor execution (Salmelin and Hari, 1994; Salenius et al., 1997; Babiloni et al., 1999) and imagery (Pfurtscheller and Berghold, 1989; Llanos et al., 2013), as opposed to an ipsilateral synchronization. For example, left hand motor imagery is known to attenuate alpha rhythmic activity in the right cortical motor area, instead of increasing alpha activity in the left cortical motor area.

The signs of the slopes α_*h,a*_ are based on the typical 2D sensorimotor alpha rhythm-based cursor control achieved by Wolpaw and McFarland (1994), which was originally developed based on the above neurophysiological findings. In such a scheme, the hemispheric lateralization of hand motor imagery is used to control horizontal cursor movement while the bilateral hand motor imagery or rest controls the cursor up or down, respectively. In other words, to control the cursor to the right (*v*_*x*_ > 0), the subject would display alpha desynchronization, or a relative decrease in alpha band power, in the left motor cortex, and vice versa. Hence, the encoding functions for *h* = “left” and *h* = “right” in the horizontal (*a* = *x*) control appear mirrored. To control the cursor upwards (*v*_*y*_ > 0), the subject should display alpha desynchronization, or a relative decrease in alpha band power, averaged across both left and right motor cortices (i.e., both-hand motor imagery). A lack of such desynchronization (i.e., rest) produces control in the downwards direction. This control over 2D space is captured by the signs of α_*h,a*_, which is preserved in other configurations. This allows subjects to input their 2D motor intention into BCIs relatively naturally by doing hand motor imagery.

##### Classic vs. centered configurations

In an ideal case, however, subjects would demonstrate SMR changes that produce orthogonal commands for all four directions, as it theoretically offers the maximal amount of differentiability in the encoding between the four directions. This is embodied by a centered configuration ideal which we also propose and include in the simulator as a less realistic but a more ideal case that would raise the BCI system's performance ceiling in the simulated environment even higher. By ensuring that *A*_*h,a*_ varies in both $v_{a}^{\prime }$ directions for all control axes *a* by the same amount, the simulated subject produces orthonormal control vectors that perfectly span a 2D control space.

A simple numerical example is useful in understanding this hypothesized effect of encoding configurations on the alpha power features of the simulated EEG. Given a pure rightwards movement intention input $\left(v_{x}^{\prime }=1,v_{y}^{\prime }=0\right)$, a classic encoding maps this intention to have a high *A*_right,*x*_ ≈ 1 and a low *A*_left,*x*_ ≈ 0, correctly resulting in a rightwards horizontal movement once the generated EEG is decoded using Equation (1). Meanwhile, the lack of vertical movement intention, represented by $v_{y}^{\prime }=0$, is mapped to a value of approximately 1 for both *A*_right,*y*_ and *A*_left,*y*_. Thus overall, the intention has lowered *A*_left_ while *A*_right_ has not changed from the no-intention state. This introduces an unwanted vertical component to the decoded velocity (an upwards component specifically, because *C*_*y*,_*t*__0__ is now less negative, Equation 1). Thus, using the classic encoding, we can expect a diagonal bias of the cursor movement toward the up-right direction, given subject intention input of pure rightwards movement.

In contrast, using the centered encoding, the same intention input $\left(v_{x}^{\prime }=1,v_{y}^{\prime }=0\right)$ results in approximately the same horizontal control signal as the classic encoding, but *A*_*right, y*_ is about 0.5 lower than *A*_*right, x*_ while *A*_*left, y*_ is about 0.5 higher than *A*_*left, x*_. Thus the intention decreases *A*_left_ by the same extent that *A*_right_ increases, eliminating the unwanted vertical component. Thus, using the centered encoding, we hypothesize that the subject would be able to control the cursor more closely to their true intention input, although the model is now assuming ipsilateral alpha synchronization during motor imagery in addition to contralateral desynchronization which may be considered a step down in realism of the resulting EEG. (However, reports of ipsilateral alpha synchronization observed during motor imagery do exist, although rarer; Pfurtscheller and Neuper, 1997). Based on this understanding, we evaluated the effect of centered and classic encoding models on the resulting task-specific SMR features (i.e., alpha powers) in the simulated EEG by conducting three individual runs of 2D target reach, in which the targets were displayed in random order at cardinal positions to prompt the subject to move the computer mouse in each direction in a straight line. To verify the effect of the classic and centered encoding in the simulated environment, we visualized the decoded trial-by-trial cursor trajectories from the 2D runs, as well as the effect on three different performance metrics (**Figure 4**) designed to evaluate the extent of the diagonal bias that is implied by the classic encoding and corrected by the centered encoding. Cursor position covariance for each run, *C*_*x,y*_, was utilized to estimate the presence of unwanted diagonal bias. True vs. decoded velocity angle is the angle of deviation between the true intended velocity vector and the decoded velocity vector actually applied to the displayed cursor. To calculate this, the simulator simply records the input velocity vector as the true intention and compares its alignment against the decoded velocity vector at each time sample of feedback control. Lastly, the trajectory length to target, which is simply the Euclidean norm of the decoded velocity summed over all time samples of feedback control, is a less direct but more practically meaningful measure of how a diagonal bias would affect BCI performance by elongating the travel distance and potentially travel time of the cursor to the target.

##### Perturbed configuration (subject-fitting of encoding functions)

Figure 2 also mentions the perturbed configuration, which is designed to fit the sigmoid functions by analyzing the EEG recorded in previously conducted target reach experiments. This is a more ambitious encoding approach that attempts to fit sigmoid curves that match the subject's C3 (*h* = “left”) and C4 (*h* = “right”) alpha band powers when presented with each of the 4-class targets. This allows the simulator's encoding of the motor imagery neural activity to be subject-specific, if the subject undergoes a 1D LR and UD or 2D LRUD reaching motor imagery experiment as a calibration session. EEG epochs from feedback control periods of hit trials in each of the four directions were extracted and pre-processed (Notch filtered 58–62 Hz, bandpass FIR filtered 2–60 Hz). Alpha powers from hit trials filtered by each direction and each control axis were averaged, resulting in 12 mean alpha power values: μ_*L*_, μ_*R*_, μ_*U*_, μ_*D*_, μ_*LR*_, μ_*UD*_ for *h* = “left” and “right”. These are proportional to average amplitudes of alpha bandpass filtered signals and therefore can serve as factors for α_*h,a*_ values used to fit the sigmoids. We do this by assigning $v_{x}^{\prime }=-1$ to μ_*L*_, $v_{x}^{\prime }=0$ to μ_*UD*_, $v_{x}^{\prime }=1$ to μ_*R*_, $v_{y}^{\prime }=-1$ to μ_*U*_, $v_{y}^{\prime }=0$ to μ_*LR*_, and $v_{y}^{\prime }=1$ to μ_*D*_, making the reasonable assumption that the EEG during vertical control would contain no horizontal control intention, and vice versa. μ values were normalized by dividing the maximum μ within each control axis, ensuring that 0 ≤ μ ≤ 1. This gave us enough data points (3 per *h, a* combination) to fit the α_*h,a*_ and *k*_*h,a*_ of the sigmoids by nonlinear regression with iterative least squares estimation. This offline pipeline, which accesses continuous recorded EEG in the BCI2000 data format and outputs the α_*h,a*_ and *k*_*h,a*_ values, is implemented as a custom MATLAB script and is available for use with the simulator, although it is not yet validated with a large group of subjects with undoubtedly vastly varying SMR activation patterns during motor imagery. Nonetheless, to both demonstrate its functionality and to increase the realism of the EEG generated, we used this pipeline to generate α_*h,a*_ and *k*_*h,a*_ values from a single high performing subject's data from one of our previous studies (Stieger et al., 2020, 2021a), and this model was used for running the simulated experiments in the parameter effect comparison study.

### Generation of scalp EEG *via* forward solution

**Figure 3:**
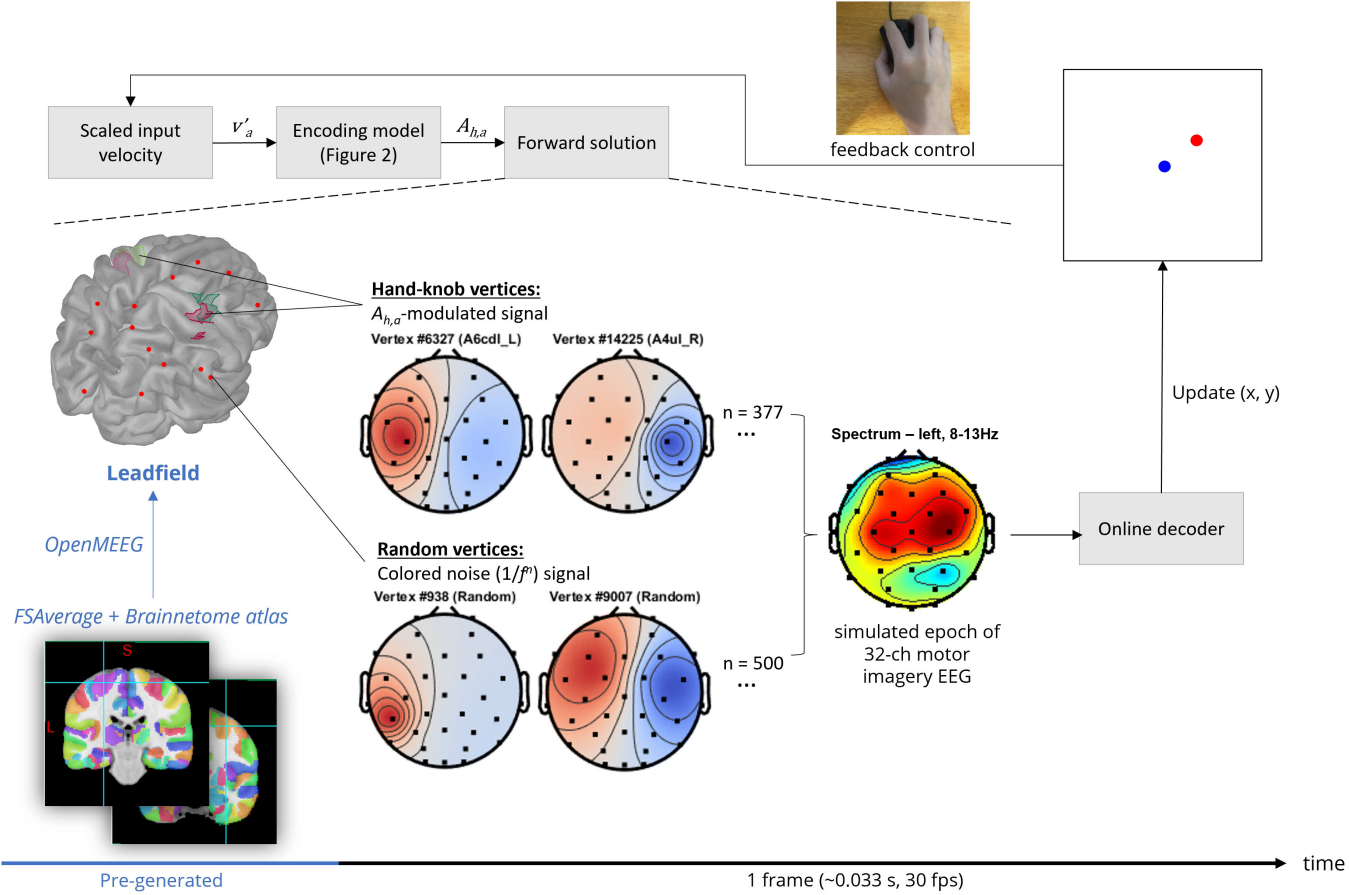
Frame-by-frame pipeline of the EEG BCI simulation (shown in black text), in addition to the pre-loaded lead field computation process (shown in blue text).

An overview of the scalp EEG generation pipeline is shown in Figure 3. OpenMEEG, a forward solver that employs a symmetric boundary element method (Gramfort et al., 2010; Kybic et al., 2005), was used to compute a lead field matrix of the dimensions 32 channels × 15,002 vertices using the FSAverage anatomy template cortical surface as the source space (Fischl et al., 1999) and the default BioSemi 32-channel MRI registration *via* Brainstorm (Tadel et al., 2011). 15,002 corresponds to the number of vertices provided by the low-resolution cortical surface provided by the FSAverage template which is sufficient for our use. Vertices located in the left and right hand-knob areas as specified in Viganò et al. (2019) were then labeled based on the Brainnetome atlas parcelation (Yu et al., 2011). Specifically, these were areas A4ul_L, A4ul_R (left and right area 4—upper limb region), A6cdl_L and A6cdl_R (left and right caudal dorsolateral area 6).

The source-level background activity was modeled as a Brownian noise (1/*f*^2^) signal (maximum amplitude 50 μV, so signal-to-noise = 100μ*V*/50μ*V*) assigned to 500 random vertices. This is the standard level of complexity in modeling background brain processes in other forward solution-based simulations (Haufe and Ewald, 2016), although the number of vertices as well as the number of unique activation signals used could serve as future hyperparameters to further increase the complexity of the background activity simulation. However, the lead field projection is a limiting step of the frame-by-frame pipeline in terms of computation time, at least in the simulator's current implementation. Therefore, the number of vertices used in the forward solution is limited by the target update rate, which was 30 frames per second on low-end computer systems for the included experimentation.

The activation signal corresponding to source-level motor imagery, in general, was modeled as four separately defined amplitude time series signals. Each signal was generated by bandpass filtering uniform white noise with a Kaiser window FIR filter with edge frequencies matching the alpha band (5–12 Hz) with transitions between 3–5 and 12–14 Hz. The filter order was automatically estimated using empirical formulae described in Oppenheim et al. (1999). Then, the resulting signal was filtered with a Tukey window with width = 0.95 times the epoch length and cosine fraction = 0.8, then its amplitude was normalized by the *A*_*h,a*_. This normalization simulates the event-related mu-rhythm desynchronization or synchronization that is associated with motor imagery as described in the previous section on encoding model design.

## Results

**Figure 4:**
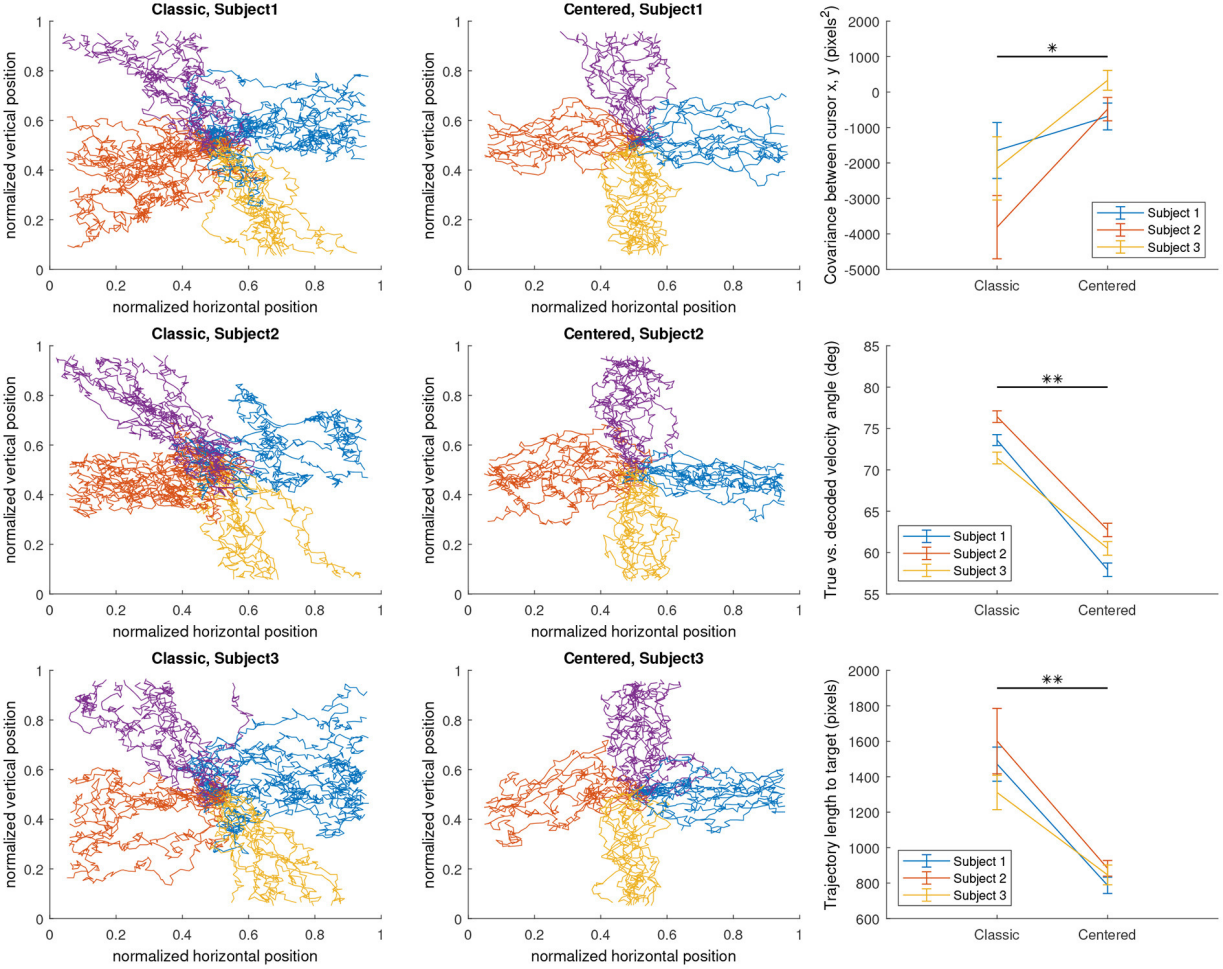
The effect of classic and centered neural activity encoding models on the decoded cursor trajectories in a 2D left/right/up/down discrete target reach task, as well as on various performance metrics on a trial-by-trial basis. Four-class targets were located in each cardinal position as a bar with a thickness of 0.0625 normalized task space units.

We found that both encoding models behaved as expected (Figure 4). The centered configuration lowered the diagonal bias relative to the classic configuration in all calculated metrics on average. In all three subjects, the centered configuration had less negative covariance between horizontal and vertical cursor positions (*p* = 0.0355); less angular deviation between true and decoded cursor velocities (*p* = 0.00258); and shorter trajectory length over time (*p* = 0.00215), all using paired *t*-tests. The statistical significance of the trends as well as the visualized trajectories indicate a clear effect of the encoding model on the decodable features of the simulated EEG. These results support our previous assertion that the simulator's well-defined encoding models allow for the isolation of decoder and task parameters as the cause of the effects in performance.

**Figure 5:**
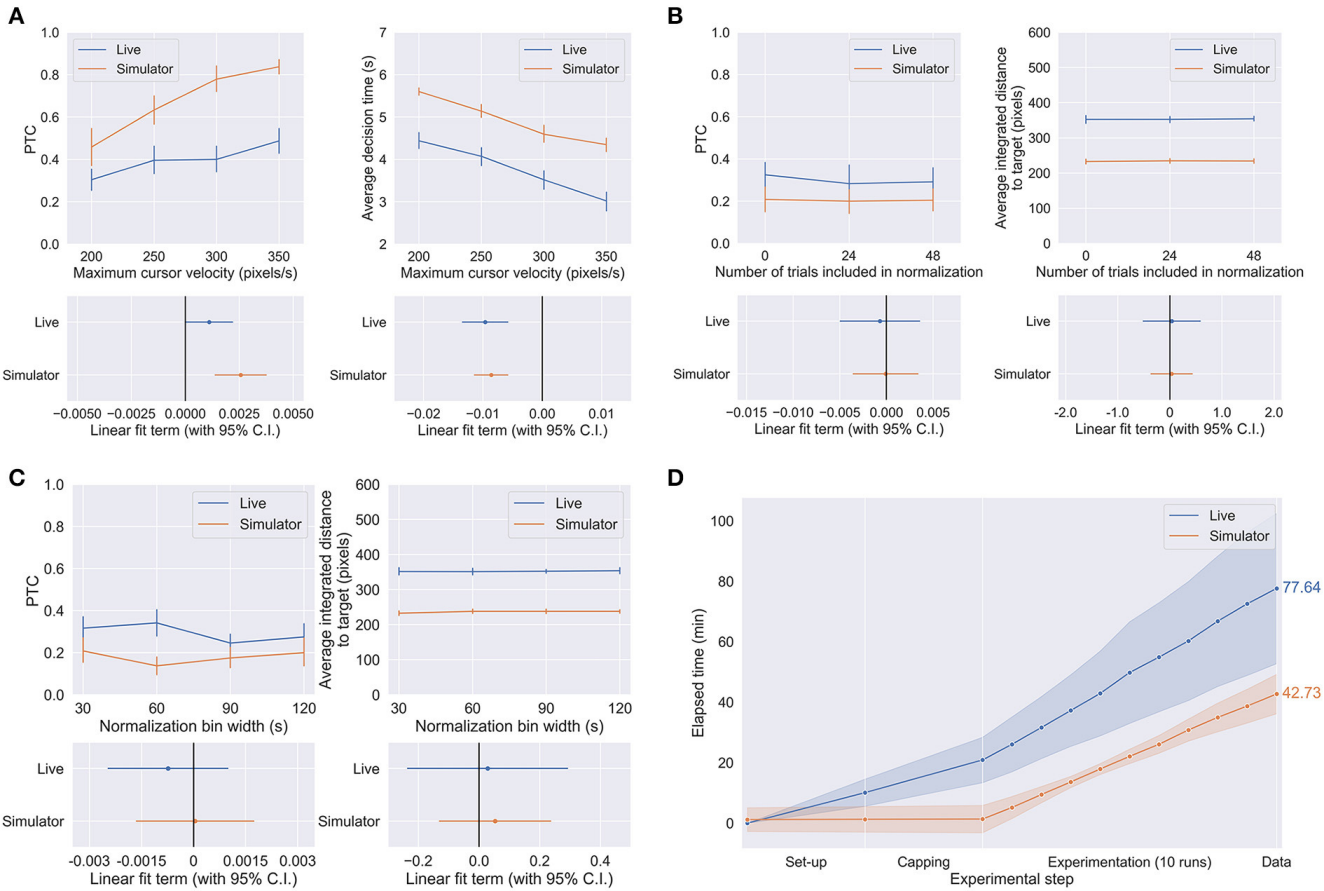
**(A–C)** The effect of CV **(A)**, NT **(B)**, and BW **(C)** on performance metrics as defined in Methods. **(D)** Time-to-data curves for the live and simulated experiments, with mean value in minutes over 10 subject sessions shown on the right. Set-up only includes necessary sanitization of the experimental venue and electrode cap, and the launching of essential experiment softwares: BCI2000 (for recording the EEG, and timing and visualizing the experiment) and g.RECORDER (for signal transmission checks and application of hardware settings such as voltage sensitivity and online noise removal). Capping includes the actual electrode capping process as well as the conduction of signal transmission checks to ensure that the EEG was being acquired and processed appropriately. Each point in the experimentation represents the end of each run, including time spent on subject-requested breaks, equipment troubleshooting, and/or providing additional instructions to the subject as necessary.

Figures 5A–C shows the performance of the human subjects in the live and simulated experiments when subject to the parameter changes described in Table 1 and Section Methods. We found that maximum cursor velocity was a significant linear predictor of PTC in both live (*p* = 0.0496) and simulated (*p* = 0.000109) conditions and average decision time (both live and simulated *p* < 0.0001). In the other trends, no statistically significant effects of NT and BW on performance were observed for either the live or simulated experiments (live PTC vs. BW: *p* = 0.396, all others: *p* > 0.5). The statistical significance is visualized in Figures 5A–C by the linear fit terms (i.e., regression coefficients) with their 95% confidence interval. The trend in each parameter-environment pair is significant if the confidence interval of its linear fit term crosses the zero line.

Figure 5D shows the average time spent on the live and simulated experiments, per phase of the experiment. We can make this comparison because the live and simulated experiments were run in parallel on the same subjects with the identical trial structure and length. Despite using a dry electrode system which is typically considered a fast and convenient option available for live EEG BCI experiments, the BCI simulator only took about 55% of the time the live experiments took to obtain the same amount of data used to conduct the same statistical analysis and to arrive at the same conclusions. The reduction in time-to-data would undoubtedly be greater in comparison to wet electrode systems. The reduction in time-to-data comes from all phases of the experiment but is most obvious in the set-up and capping phases, which are completely unnecessary in the case of the simulator. The simulator does require set-up in the sense of initialization of system parameters, but the same can be said for the experiment software involved in running the live experiments and was therefore excluded from this comparison.

## Discussion

The present study had three major goals: (1) to develop a motor imagery EEG BCI simulator, which is novel in closed-loop BCI simulation and can serve as an adoptable and readily extendable framework for future simulated EEG experiments; (2) to demonstrate the proposed simulator's ability to conduct pseudo-online BCI experiments to replace or supplement offline and online experimentation in the future, and the merits in doing so, and; (3) to do such validation by testing parameters of practical interest to motor imagery BCI studies.

We achieved the first objective by ensuring that each step of our simulation pipeline was congruent with well-established standards in offline EEG simulation that increase the realism of the EEG generated and the adoptability of the resulting method. For example, we use a symmetric boundary element method with a realistic anatomical human head model template, as well as the option to input custom head models. In the forward solution, one dipole is assigned to each vertex with its orientation perpendicular to the cortical surface, which is a standard in many lead field calculation processes. Furthermore, our approach is open to customized source locations, activation signals, and orientations, effectively allowing vastly different kinds of EEG to be generated in real-time for different purposes—although new encoding models would need to be developed for paradigms beyond 2D motor imagery. We also proposed various encoding model configurations to close the loop between motor intention and EEG, a feature not available in offline simulations. These were developed based on reasonable assumptions grounded in known motor imagery neurophysiology (see Section Methods).

We achieved the second objective by running a parallel study involving live and simulated experiments with as many factors (subjects, task, decoder) kept constant as possible. By doing so, we showed that the simulator led to the same hypothesis testing results on the effects of the parameters as the live experiments did. We also showed that the simulator spent much less time than the live experiments to collect the same amount of data used to reach the conclusions, demonstrating the simulator as a rapid prototyping test-bed for BCI systems. More qualitative advantages of the simulator that we have demonstrated include the abilities to: conduct experiments remotely and without dedicated EEG equipment; record ground truth intention over time, which allowed us to evaluate metrics such as true vs. decoded velocity angle deviation which may be of interest in BCI behavioral studies; test BCI system components in controlled environments with customizable levels of session and subject variability (by changing the encoding function parameters) as well as signal-to-noise conditions (by adjusting the maximum amplitudes of source-level task-related vs. background activation signals). All of these advantages overcome some innate challenges experienced by live BCI experimentation, and it is our hope that the simulation framework provided by our study encourages more active efforts by researchers to evaluate and improve their decoding algorithms and tasks in online and pseudo-online environments.

Lastly, in fulfilling our third objective, we found interesting results regarding the tested parameters that warrant further investigation.

### Maximum cursor velocity

We had originally hypothesized that the effect of maximum cursor velocity on PTC would vary between subjects depending on average subject performance. This was due to vastly different levels of SMR control consistency observed between subjects (that depend on their BCI training and experience, as well as their mental state). We hypothesized that subjects with highly consistent SMR control and therefore higher average performance would display linearly increasing performance with increasing maximum cursor velocity, while subjects who had poor consistency in their SMR activity and therefore control of the cursor would display an inverse U-shaped trend of performance against maximum cursor velocity, due to the higher number of wrong-target hits (usually called “misses,” but such trials were included in our analysis as markers of poor performance, see Sections Methods and Results) by uncontrollably fast moving cursors. The idea would be that increasing the maximum cursor velocity (and by extension the frequency of decisions) would decrease the accuracy for such subjects who are unable to exhibit sustained intention, before reaching a critical point where only the initial correct intention is captured. Such a U-shaped trend would also have been consistent with studies that attempted to optimize cursor velocity, i.e., gain (although different from maximum cursor velocity in this study) in 1D motor imagery cursor control (McFarland and Wolpaw, 2003; Willett et al., 2019).

Instead, we found that increasing the maximum cursor velocity increased PTC regardless of the subject's average PTC. While this result may appear contradictory to our hypothesis, they are certainly not irreconcilable. We suspect that there is a separate confounding factor at play that is highly related to (but perhaps not linearly correlated with) maximum cursor velocity. We suggest two possibilities for this: the subject's level of cognitive engagement in the task, and the dynamics of cursor position error. The former refers to the idea that if a cursor is able to move at higher speeds, the task engages the subject to pay more attention to the task due to the fact that there is now less time to correct for errors if the cursor moves off in the wrong direction. In this case, it is also easy to see why it might be related to maximum cursor velocity rather than a constant gain on cursor velocity - the unknown increases in the speed at which the cursor can potentially move may, on average, increase the task difficulty and boost the subject's engagement. Alternatively, the engagement level may have increased because trials now take a shorter period of time on average, as supported by our results. The latter refers to the possibility that higher cursor speeds may have increased performance by reducing the temporal delay between the subject's true intended cursor position/trajectory (as visualized in the subject's mental space or internal coordinates, etc.) and the updated cursor position/trajectory on the screen provided as visual feedback. Indeed, in control theory, delays are regarded as a major cause of instabilities and suboptimal limits in control performance, and they are usually compensated for by complicated controllers that estimate delay elements (Mirkin and Palmor, 2005; Brown and Coombs, 2008). While our brain can undoubtedly learn to correct for such visual input delays in much more sophisticated ways than man-made controllers, a need for it to do so would worsen average BCI control performance and training times. Therefore, it would be helpful for BCIs if some optimization on the maximum cursor velocity could, to some extent, eliminate the delay element that enters the closed-loop system in the first place.

Furthermore, Willett et al. (2016) showed quantitatively that increasing cursor velocities prompted subjects to successfully modulate their own control signals to be shorter, supporting our results. Combining these findings and also our previous study (Shin et al., 2021), our results suggest that relaxing maximum cursor velocities in general could improve performance and control efficiency for continuous cursor control tasks.

### Z-score normalization bin width

We had hypothesized that increasing Z-score normalization bin width would have a positive effect on performance: a longer bin width would leave a longer time history of raw control signals to act as a statistical buffer for the incoming raw control signal to be normalized against, thereby increasing the quality of baseline SMR pattern estimation, and by extension, control signal estimation. Similarly, we had hypothesized a similar statistical robustness effect of the number of trials for control coefficient calculation.

However, we found no significant difference in performance between the values tested for both of these parameters in both the live and simulated environments. A possibility is that shorter bin widths benefited performance by increasing the recency of baseline SMR pattern estimation, to an extent sufficient to even out with the hypothesized gain in performance. Indeed, from a practical perspective, recording artifacts such as sudden movements and eye blink artifacts, when not appropriately corrected for online, may enter the normalization buffers as large spikes in amplitudes: shorter bin widths and number of normalization trials may have worked to ensure that such information that is harmful to the control signal statistics is flushed out relatively quickly before affecting online control for extended periods of time. If this is indeed the case, it would be an interesting future study to implement sophisticated online artifact removal algorithms (Kobler et al., 2020), which are actively being developed in recent years, and seeing if the effect of these two parameters is changed. An alternate explanation could be that the dynamics of this task were stable across the shortest buffer width, and therefore it had no significant effect on task performance.

### Study limitations and future work

The limitations of this work can be divided into that of the simulator, and that of the experiments conducted. As for the proposed simulator, there are clear areas for improvement. One is regarding the realism of the EEG generated. By using well-defined encoding models, we have ensured that the generated EEG varies continuously according to continuous intention input, closing the feedback loop that is neglected in offline analyses. While this allows valid closed-loop experiments to be conducted with the simulator in the implemented experimental paradigms (1D and 2D motor imagery cursor control with alpha power estimation), the EEG generated cannot be regarded to be equivalent to real EEG in general, and it cannot be expected to work with paradigms outside this family of task-decoder combinations. Despite this, the simulation of EEG under other paradigms may be achieved by extending the current simulator. The proposed approach, much like other available ground-up EEG simulations, requires users to design a parameterization method of EEG and a neural activity encoding model that fits their purpose of use. The alternative would be the use of endpoint-focused approaches to EEG simulation, such as generational adversarial networks (GANs) and variational autoencoders (VAEs) (Zhang and Liu, 2018; Aznan et al., 2019; Bao et al., 2021; Fahimi et al., 2021; Kunanbayev et al., 2021; Ko et al., 2022) and waveform decomposition and reconstruction-with-noise techniques (Yeung et al., 2004; Lotte, 2011; Bridwell et al., 2016; Dinarès-Ferran et al., 2018). Such approaches pay less attention to ensuring that the generative model is consistent with our understanding of the origin of the EEG and its features, or the neurophysiology of BCI control, in exchange for highly realistic EEG. Among these, VAE-based approaches may be especially well-positioned to overcome the aforementioned limitation of multi-class EEG generation by establishing a continuous parameterization subspace from training epochs of EEG. However, the parameterization only seeks to capture the statistics of real training data, and thus lies outside the task space. Instead, in the future, it may be possible to implement a closed-loop decoder-encoder framework such that a training epoch of EEG is decoded into continuous parameter(s) that represent intention variable(s) in the task space, which is then fed into the encoder of a generative model to generate continuously varying EEG. Alternatively, a dynamical systems perspective on motor control (Shenoy et al., 2013) may provide hints for a simulation method that does not rely on encoding models to parameterize intention. In this case, the challenge would be implementing a time-varying generative model that work toward implicit intention “output(s)” based on past perturbations to the system, and ensuring its function in a closed-loop environment.

While human experimentation should definitely remain as the ultimate environment for drawing scientific conclusions (especially those relating to BCI-relevant neurophysiology) and holistically validating BCI systems, closed-loop simulation is an interesting framework that has been demonstrated by us and others to be able to serve as a controlled environment for rapid testing of large arrays of parameters as well as initial evaluations of decoder and task designs with feedback control. In addition, as explained previously, our closed-loop simulation offers merits such as the availability of continuous true intention recording, ability to assess a decoding algorithm's performance ceilings (if noise conditions in the simulation settings are minimized), as well as increased accessibility for both subjects and experimenters to online EEG experiments. However, this study did not specifically aim to exploit or demonstrate these advantages, as our priority was to assess the experimental functionality of the simulator at a basic level. Future studies should perhaps do so. In particular, the simulator is particularly well-positioned to answer some important behavioral questions in the motor imagery BCI context, such as investigating how subjects behave in various virtual environments with different object interactions, rules, target, and cursor sizes.

Another notable feature of the simulator that was not rigorously examined in this study is the ability to quantitatively control noise conditions or neural activity perturbation. As mentioned in Section Methods, the simulator is equipped with tools to control the signal-to-noise ratio (SNR) at the source level, and perturb the neural encoding model manually or by fitting to a subject's previously recorded EEG data. The signal-to-noise ratio parameter in particular could, in the future, undergo optimization to result in cursor control behavior closest to the live experiments. This could be challenging due to the difficulty in defining the true or observed SNR from live EEG data to serve as a standard. For example, we could reasonably estimate live SNR in our case to be the alpha power in C3/C4 divided by the average non-alpha power in all non-C3/non-C4 channels. However, a value resulting from such a definition would not be directly comparable to the SNR parameter in the simulator as it is defined currently, although we may argue that they are at least comparable in scale. A low SNR configured for the simulator artificially lowered the performance in the simulated experiments, as observed by the lower PTC and longer decision time on average compared to live under several experimental conditions. In the simulator, other types of perturbations can also be modeled in the given framework e.g. display latency. Future studies could use such approaches to test subject behavior and/or decoder performance when subject to such conditions designed to model real-life challenges to stable BCI system usage.

As for the study, a limitation was that the calibration trial did not provide enough time for the normalization bin to fill up initially, possibly leading to a diluted effect of the BW parameter on the performance metrics which were evaluated across all trials. While this short calibration protocol was chosen to be in line with literature using the autoregressive power spectral density estimation (Edelman et al., 2019; Meng et al., 2022) which build the normalization bin during online control, it did restrict the number of trials in which the BW had statistical influence. A future study could perhaps be designed to confirm the effects of BW on motor imagery BCI with a larger subject and trial pool.

## Conclusion

In summary, we have developed a motor imagery EEG BCI simulator that provides a framework for closed-loop motor imagery EEG generation. Inspired by a variety of forward solution-based offline EEG simulators, online neural activity simulation studies, and motor imagery studies, our framework contributes toward promoting the online testing of motor imagery BCIs and noninvasive BCI systems. We demonstrated the usage of our simulator in gaining insights into the effect of several important decoder and task parameters, including the limit on cursor velocity as a parameter, which we found to significantly affect performance. We believe that the adoption and improvement of such software, including ours, will lower the barrier to online experimentation and ultimately accelerate the development of noninvasive BCI decoders, tasks and systems that are closer to real world applications.

## Data availability statement

The data collected and analyzed in this study are available in the Figshare repository at: https://doi.org/10.6084/m9.figshare.20383716. The code used in this study is available at https://github.com/mcvain/bci-simulator.

## Ethics statement

The studies involving human participants were reviewed and approved by the Institutional Review Board of Carnegie Mellon University. The patients/participants provided their written informed consent to participate in this study.

## Author contributions

HS, DS, and BH developed the concept, designed the experiment, and edited the manuscript. HS developed the software, acquired and analyzed the data, and drafted the manuscript. All authors contributed to the article and approved the submitted version.

## Funding

This work was supported in part by NIH AT009263 and EB021027, and DARPA HR001118S0029-N3-FP-019.

## Conflict of interest

The authors declare that the research was conducted in the absence of any commercial or financial relationships that could be construed as a potential conflict of interest.

## Publisher's note

All claims expressed in this article are solely those of the authors and do not necessarily represent those of their affiliated organizations, or those of the publisher, the editors and the reviewers. Any product that may be evaluated in this article, or claim that may be made by its manufacturer, is not guaranteed or endorsed by the publisher.
